# The Antagonistic Effects of Belladonna and Opium

**Published:** 1874-02-15

**Authors:** H. Wardner

**Affiliations:** Cairo, Ill.


### MEDICAL EXAMINER.

A

*Semi-Monthly Journal of Medical Sciences.*

EDITED BY N. S. DAVIS, M.D., AND F. H. DAVIS, M.D.

No. IV. CHICAGO, FEBRUARY 15TH, 1874. VOL. XV.

### Original Communications.

### THE ANTAGONISTIC EFFECTS OF BELLADONNA AND OPIUM.

READ BEFORE THE ALEXANDER COUNTY MEDICAL SOCIETY, DECEMBER 16TH, 1873, BY H. WARDNER M.D., CAIRO, ILL.

THE idea of neutralizing the poisonous effects of opium and its preparations by the administration of belladonna, was first brought prominently before the profession by Dr. W. F. Norris, in the October number of the *American Journal of Medical Sciences* for 1862.

There had been some cases of opium poisoning recorded, in which the free administration of belladonna, instead of increasing the narcotism appeared to supersede the action of the former on the system by substituting its own, so that, although given in quantities that would, under ordinary circumstances, be dangerous to life, the result of the antagonistic or combined action of the two poisons was the saving of human life, which would have been lost under the influence of *one*.

In reviewing the reports of cases, it is satisfactory to note that all are claimed to have been successful, save one reported by Dr. J. P. Chesney, who has the temerity to publish an unsuccessful case in the person of an infant of eight months, that had swallowed a grain of morphia four hours before medical aid was obtained. He used the cold douche; agitation; and tincture belladonna, in five drop doses, repeated at short intervals, which had the effect to dilate the pupils; but the case proved fatal at the expiration of six hours after taking the poison.

The November number of the *Chicago Medical Journal* for 1867, contains the report of a case which, when first seen, was in a state of impending death. Atropine, two grains to the ounce of water, was given hypodermically, using three drachms at once, and repeating the injection twice. Conciousness was restored, when six grains of the solid extract of belladonna were given within six hours. The patient recovered from the effects of both poisons; but there was a complete loss of sight for eighteen hours from the effects of the antidote.

Dr. Marfit (*Half-Yearly Compendium*) reports a case in which the patient took ten drachms of Magendie's solution. An emetic was given within half an hour. The patient was seen by the doctor three hours later. He would answer questions, when roughly agitated and spoken to. Half a grain of atropine was at once given by the hypodermic method. External heat applied to the limbs; and coffee and brandy freely used. When he began to rally, one-sixth of a grain alc. ext. belladonna was given every four hours until the following day, when the recovery was complete.

The same authority also gives a report by Dr. Samuel Frank, of Philadelphia, of a case in which one-eighth of a grain of atropine was administered hypodermically to a man who had swallowed an half ounce of laudanum. The results were satisfactory.

Dr. E. A. Clark, in the *Medical Archives* for January, 1869, reports the case of a man brought into the hospital at St. Louis, in a comatose condition, after having taken *one* ounce of tincture opium. The battery was used to no purpose. The man was failing, when the doctor injected one-sixteenth of a grain of atropine, and repeated the dose in twenty minutes, and again in thirty minutes. The pupils soon began to dilate, and recovery followed without further treatment.

Dr. B. J. Wilson, in the *Journal of Materia Medica* for December, 1869, reports a case of narcotism by an overdose of morphia, hypodermically administered. The case was treated by the injection of *one-fourth* of a grain of atropine. This was followed by symptoms of the *toxical effects* of *belladonna*. The case finally recovered.

A case is reported in the *Medical and Surgical Reporter* of February 20th, 1869, of a woman aged forty-five, who had taken fifteen grains of opium. An emetic was given one hour afterwards, which was followed by twenty drop doses of the fluid extract of belladonna every half hour, until two drachms were given, and the pupils had dilated to their natural size. She was then left to sleep six or eight hours, when she was well.

In THE MEDICAL EXAMINER for August, 1870, may be found the report of a case of poisoning, in a man, by twelve grains of opium. When first seen by the doctor (T. Griffin), all sensation was gone; breathing three or four times per minute. A solution of atropine, one grain to the ounce was given, hypodermically, in small doses, every fifteen minutes, until one twenty-fourth of a grain was administered. The symptoms soon began to change, and the man recovered in nine hours after swallowing the poison.

Dr. Bucklin, in the *New York Medical Journal* for October, 1871, reports a case of poisoning by opium, in which death seemed inevitable, where, as a last resort, twenty drops of Flemming s (?) solution of atropine was poured into the patient's mouth, and run down his throat, as he could not swallow. This was repeated twice, at intervals of half an hour. In four hours he was convalescent, and recovered, the dilation of the pupils lasting one week.

I have noted the reports of ten other cases of opium poisoning, all of them in a condition of impending death, which were successfully treated by belladonna as the principal agent, other means being used as deemed appropriate to each case.

I have recently had two cases. One, a man aged fifty-four, took twelve grains of morphia, for the avowed purpose of suicide. I saw the case about two hours after the poison was swallowed. Anæsthesia was perfect; the passing of the finger into the fauces and about the epiglottis, the handling of the naked eye-ball, and rough agitation, produced no effect. The breathing was heavy and labored; the face livid; lips purple; and the pupils had contracted to their smallest capacity. There was perfect cessation of all muscular action, except in those muscles connected with the circulation of the blood and respiration; and they were evidently slowly failing. The cold douche, was freely applied to the head, and one twenty-fifth of a grain of atropine given subcutaneously. This was repeated in fifteen minutes, and again in thirty minutes. There was yet no improvement. A little of the atropine solution was dropped into his right eye. At the expiration of fifteen minutes, there was a little dilation of the left pupil. External heat was applied about the limbs, which were growing cold. At the expiration of forty-five minutes from the last injection it was repeated, in a little larger dose, so that he got, in all, about one-fifth of a grain of the atropine. Shortly after the last injection, the pupil of the eye treated was well dilated; his symptoms changed; he became warm, and was soon in a profuse perspiration. A large quantity of water was now passed through the catheter. A little action of the eyelids was observed, when the eye was handled. The return to semi-consciousness soon followed; and in nine hours he was able to answer questions in monosyllables. The pupil of the right eye was largely dilated, until after the second day; the left pupil was not unnaturally dilated at any time. He fully recovered from the effects of both poisons in forty-eight hours. But he suffered from some severe burns on his feet, legs, and hands, caused by application of hot bricks, and bottles of hot water, made by his friends, who now frequently remind him that his sufferings from the burns are but a foretaste of what he was about to endure in the hereafter.

Another case was that of a "sporting woman," who had swallowed, as near as could be estimated, about seven grains of morphia. The treatment was, in all respects, similar to that of the above case, except that only one-eighth of a grain of atropine was given in all, and she was not burned by the hot applications. She will walk the earth awhile longer.

Of the manner in which the atropia acts in these cases, there may be different theories. It is, most probably, that of a diffusible stimulant, having an influence over certain nerve-centers which supersedes that of opium. Recent experience goes to show that it is also useful in restoring the functions in other cases of collapse occurring suddenly from various causes, as cholera, chloroform, veratrum viride, or any agent capable of producing paralysis of the muscles of the heart and respiration.

Of the treatment of poisoning by belladonna, and stramonium, a few cases are recorded, in which opium has been equally successful when used as an antidote to these toxical agents.

Dr. Sinio, in the *Bulletin de Therapeutique*, reports a case of poisoning by a decoction of belladonna leaves, successfully treated by the use of laudanum, in frequent, full, medicinal doses.

The *Half-Yearly Compendium*, part 3d, page 48, gives the case of a colored boy, who, by mistake, took one-eighth of a grain of atropia, followed by the symptoms of belladonna poisoning. One-half hour afterward, one-fourth of a grain of morphia was given in an half ounce of whisky, and repeated in fifteen minutes, and again in one hour, and also in one hour and a half, from which time he was relieved and made a good recovery. The same authority gives the case of a patient poisoned by one grain of atropia. He was seen, by Dr. Fox, in twenty-five minutes, who treated him by hypodermic injections of one-eighth grain of morphia, every ten minutes, until the delirium was controlled, and he dropped into a quiet sleep, from which he awoke without a bad symptom.

The *Chicago Medical Journal* for December, 1868, gives the report of a case of a mother and two children, poisoned by drinking a decoction of stramonium seeds. They were treated successfully by laudanum and morphine.

Dr. Bernhard Kavanaugh saw two children, aged two and three years, in July, 1869, poisoned by fluid extract belladonna. They were treated by stomach-pump, and the injection of three and five drops of laudanum respectively, repeated every hour during the night. At the expiration of two days both had recoved.

Another case is reported by Dr. Drake, of Iowa, in which a woman took fifteen grains of extract belladonna. She was successfully treated by half grain doses of morphia every hour, until the pupils began to contract, when the intervals between the doses were gradually lengthened until reason was restored, which was in fifteen hours.

A case is also given by Dr. C. Johnson, of Baltimore, which was of poisoning, by two-thirds of a grain of atropia. It was successfully treated by the hypodermic use of morphia, aided by the stomach-pump, whisky, caffeine, and the battery.

There is also a case of poisoning by belladonna, used as a vaginal injection for the purpose of producing an abortion, reported in the *Michigan University Journal*, which was soon relieved by vaginal washes of laudanum, and small doses of the same, frequently repeated.

In the preceding extracts of reports it will be observed that those patients to whom was administered the larger doses of atropia as an antidote, suffered from the toxical effects of that drug after it had overcome the narcotisim, and that the smaller doses, frequently repeated, produced the most satisfactory results. It is to be regretted that those cases in which atropia has failed as an antidote have not been reported, if such there have been; and it is most probable there has been more than *one* failure. As the subject now presents itself, scarcely a person should die from narcotic poisoning, if a physician is called while yet there is breath and any heart action remaining.

For an explanation of how one of these poisons should overcome the other, we must look rather to therapeutics than chemistry.

Drs. Mitchel, Keen and Morebaun, after a series of experiments and observations in the hypodermic use of alkaloids, remark that "Atropia has no power to relieve pain, while morphia has; and the nearer it is injected to the seat of the pain, the greater its power; and atropia has no power to lessen the action of the morphia in this respect. Morphia does not materially affect the frequency of the pulse, while atropia accelerates it, after the first ten minutes, from twenty to fifty beats per minute; and morphia has no power over atropia in this respect. The change of the pulse is accompanied by little, if any, change in the breathing. As regards the pupil of the eye, we know their effects are mutually antagonistic; but the effects of atropia are much more durable. The cerebral symptoms produced by one alkaloid may, to a great extent, be superseded by the other; but in consequence of the difference of speed of action, and the longer continued action of the atropia, it is difficult to obtain a perfect neutralization of effects."

Morphia causes death by the paralysis, first, of the nerves of voluntary, and then of involuntary, motion. The heart gradually loses its power; the capillary circulation fails in the extremities; the respiration becomes less frequent; and the blood is surcharged with the effete materials resulting from disintegration. Atropine, now, has the effect to stimulate the action of the heart, and restore the circulation; the organs of excretion, consequently, resume their work, and recovery follows.

As a rule for the use of atropia as an antidote to morphia, we may give, as a dose, one-fortieth to one-twentieth, every fifteen to thirty minutes, until the therapeutic effects are observed. As the pupils dilate, the narcotism will disappear; and, on the contrary, the wild delirium of belladonna and stramonium poisoning will disappear, and the nervous excitement subside as the pupils contract under the antagonistic influence of opiates.

